# Work system factors affecting COVID-19 PPE use: A human factors approach to analysis of video recordings of emergency department clinical work

**DOI:** 10.1017/ash.2022.70

**Published:** 2022-05-16

**Authors:** Esosa Nosakhare, Shawna Perry, Susan Peterson, Frankie Catalfumo, Kelly Osei, Kelly Osei, Kelly Osei, Kelly Williams, Maia Bradley, Marium Sultan, Oluseyi Daodu, Nivedha Prabhu, Ayse Gurses

## Abstract

**Background**: The effectiveness of PPE in preventing self-contamination of healthcare workers (HCWs) and transmission of pathogens (airborne and contact) in the emergency department (ED) is highly dependent on consistent, appropriate use of and other interactions (eg, storing, cleaning, etc) with the PPE. Pre–COVID-19 studies focused primarily on individual HCW contributions to incorrect or suboptimal PPE use. We conducted an analysis of ED video recordings using a human-factors engineering framework (ie, The Systems Engineering Initiative for Patient Safety, SEIPS), to identify work-system–level contributions to inappropriate PPE usage by HCWs while they provide care in their actual clinical care environment. **Methods:** In total, 47 video sessions (each ~15 minute) were recorded between June 2020 and May 2021 using a GoPro camera in an 8-bed pod area, designated for persons under investigation (PUI) and confirmed COVID-19–positive patients, in an ED of a large, tertiary-care, academic medical center. These recordings captured a ‘landscape view’: 2 video cameras were set up to capture the entire ED pod area and HCWs as they provided care. A team with hemorrhagic fever expertise, infection prevention and control expertise, and ED expertise reviewed each video together and extracted data using a semistructured form. **Results:** Guided by the 5 components of the SEIPS work system model, (ie, task, physical environment, person, organization, tools and technology), multiple work system failure points influencing HCWs appropriate use of PPE were identified. For example, under the task component, HCWs were observed not doffing and donning in recommended sequence. Also, inconsistencies with COVID-19 status signage on a patient’s door and ambiguous labelling of work areas designated as clean (donning) and dirty (doffing) sites acted as a barrier to appropriate PPE use under the physical environment section. **Conclusions:** Human factors–based analysis of video recordings of actual ED work identified a variety of work system factors that impede appropriate or correct use of PPE by HCWs. Future efforts to improve appropriate PPE use should focus on eliminating or mitigating the effects of these work system factors.

**Funding:** US CDC

**Disclosures:** The authors gratefully acknowledge the CDC for funding this work. This material is based upon work supported by the Naval Sea Systems Command (under contract No. N00024-13-D-6400, Task Order NH076). Any opinions, findings and conclusions or recommendations expressed in this material are those of the author(s) and do not necessarily reflect the views of the Naval Sea Systems Command (NAVSEA) or the US CDC.

